# Acetic acid stress in budding yeast: From molecular mechanisms to applications

**DOI:** 10.1002/yea.3651

**Published:** 2021-05-27

**Authors:** Nicoletta Guaragnella, Maurizio Bettiga

**Affiliations:** ^1^ Department of Biosciences, Biotechnology and Biopharmaceutics University of Bari A.Moro Bari Italy; ^2^ Institute of Biomembranes, Bioenergetics and Molecular Biotechnologies National Research Council Rome Italy; ^3^ Department of Biology and Biological Engineering Chalmers University of Technology Gothenburg Sweden; ^4^ Bioeconomy Division EviKrets Biobased Processes Consultants Landvetter Sweden

**Keywords:** acetic acid stress, cell factory, industrial biotechnology, *Saccharomyces cerevisiae*, signalling, yeast

## Abstract

Acetic acid stress represents a frequent challenge to counteract for yeast cells under several environmental conditions and industrial bioprocesses. The molecular mechanisms underlying its response have been mostly elucidated in the budding yeast *Saccharomyces cerevisiae*, where acetic acid can be either a physiological substrate or a stressor. This review will focus on acetic acid stress and its response in the context of cellular transport, pH homeostasis, metabolism and stress‐signalling pathways. This information has been integrated with the results obtained by multi‐omics, synthetic biology and metabolic engineering approaches aimed to identify major cellular players involved in acetic acid tolerance. In the production of biofuels and renewable chemicals from lignocellulosic biomass, the improvement of acetic acid tolerance is a key factor. In this view, how this knowledge could be used to contribute to the development and competitiveness of yeast cell factories for sustainable applications will be also discussed.

## Take Away

- Acetic acid stress is a frequent challenge for budding yeast.
- Signalling pathways dissection and system‐wide approaches reveal a complex picture.
- Cell fitness and adaptation under acid stress conditions is environment dependent.
- Tolerance to acetic acid is a key factor in yeast‐based industrial biotechnology.
- There is no ‘magic bullet’: An integrated approach is advantageous to develop performing yeast cell factories.

## Graphical abstract

Multiple factors influenced acetic acid stress and tolerance in the budding yeast *Saccharomyces cerevisiae*.

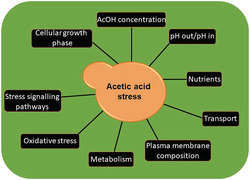

## 1 INTRODUCTION

Acetic acid stress represents a frequent challenge to counteract for yeast cells under several environmental conditions and industrial bioprocesses. In the budding yeast *Saccharomyces cerevisiae*, acetic acid is a normal co‐product of alcoholic fermentation; thus, cells under physiological conditions do not normally sense this compound as toxic and can use acetate as a regular carbon source by channelling it into respiratory metabolism. On the other hand, extracellular acetic acid can be an environmental challenge and trigger adaptive responses at sublethal concentrations or alternatively be toxic and leading to cell death. In a physiological scenario where acetic acid derives from cellular metabolism, its accumulation occurs in parallel with glucose consumption and became maximal in stationary‐phase yeast cells. In a stress‐promoting context, the final toxicity for the cell is a combined function of extracellular acetic acid concentration, extracellular pH and consequent intracellular accumulation due to its uptake (Casal et al., 1996). The mechanisms underlying acetic acid stress response have been mostly clarified in laboratory yeast strains. Results may differ depending on experimental settings, including strains used, growth conditions (pH of the medium and composition), concentration of acetic acid and time of exposure. This review will highlight the knowledge concerning acetic acid stress response in budding yeast focusing on its molecular mechanisms involving cellular transport, pH homeostasis, metabolism and stress‐signalling pathways. This knowledge represents a precious resource for industrial biotechnology, where the improvement of acetic acid tolerance affected by other inhibitors like furfural and hydroxymethylfurfural is a key factor for fermentation processes using lignocellulosic raw materials as substrates. In this view, how this knowledge could be used to contribute to the development of cell factories for sustainable applications will be also discussed.

## 2 MOLECULAR MECHANISMS OF ACETIC ACID STRESS RESPONSE

Cellular transport, pH homeostasis, metabolism and stress‐signalling pathways represent overall the main factors affecting yeast response to acetic acid stress: how much extracellular acetic acid is transported into the cell, how much acetic acid will enter the metabolism and which is the threshold level of acetic acid tolerance? Answers to these questions will provide the final concentration of acetic acid in the cell and explain the activation of tolerance or toxicity mechanisms.

### 2.1 Cellular transport

Transport of acetic acid inside the cells has to be considered within the environmental context, in terms of extracellular pH and specific nutrient‐related conditions, such as the carbon source and medium composition, minimal or rich. In addition, acetic acid transport processes are also dependent on cellular growth phase. In glucose‐repressed cells, when the extracellular pH is below acetic acid pKa (4.76), it exists mainly in the undissociated form, which can freely diffuse through the plasma membrane with a rate that depends on the membrane solubility of the molecule (Casal et al., 1996). A passive diffusion facilitated by the aquaglyceroporin Fps1 has also been proposed for acetic acid by Mollapour and Piper, although this might be condition dependent (Mollapour & Piper, 2007). When the extracellular pH is higher than 4.76, acetic acid is present mainly as acetate anions, entering the cells through the two main electroneutral proton symporter, Jen1 and Ady2 (Casal et al., 2016). Both Jen1 and Ady2 transporters have also been suggested to be involved in the export of monocarboxylates, yet it is unclear how these efflux processes are regulated (Alves et al., 2020, and references therein).

Biophysical and chemical properties of the plasma membrane can also affect cell permeability to acetic acid, which itself can cause modifications in the plasma membrane composition. The amount of acetic acid entering the cells and the rate of its diffusion are partially related either to the structure of the plasma membrane, particularly to lipid composition and cell wall assembly (Berterame et al., 2016; Lindberg et al., 2013; Mira, Palma, et al., 2010; Palma et al., 2018) or to the partitioning of selected compounds, such as ethanol and *n*‐butanol, into the plasma membrane (Lindhal et al., 2017). At this regard, the ABC transporter Pdr18 has been reported to play a role in ergosterol homeostasis contributing to counteract acetic acid‐induced changes in lipid composition and permeability (Godinho et al., 2018).

Once inside the cells, acetic acid in the more alkaline environment of the cytoplasm dissociates into acetate ions and protons, causing acetate accumulation and intracellular acidification. This can lead to both inhibition of growth and metabolic activity, such as glucose consumption, in addition to other deleterious effects, including oxidative damage and energy depletion, depending on acetic acid concentration, intracellular acidification and glucose availability (Mira, Palma, et al., 2010; Pampulha & Loureiro‐Dias, 1989). A detailed model for the dynamic dependence of biomass yield and growth rate on pH conditions, acetic acid and extracellular glucose concentrations has been provided by Kitanovic et al., indicating that intracellular acidification due to accumulation of dissociated acetic acid in the cytosol is required for acetic acid toxicity, which creates a state of energy deficiency and nutrient starvation (Kitanovic et al., 2012). This study points the attention on the strict relationship between acetic acid tolerance, environmental context and cell growth phase. Increased acetic acid resistance has been observed in exponential cells treated with the same concentration of acetic acid but grown in glucose limiting or de‐repressing conditions (Guaragnella et al., 2013). Accordingly, yeast cells in diauxic shift after glucose exhaustion or in stationary‐phase, are much more tolerant to acetic acid at low pH compared to exponential cells (Ludovico et al., 2002).

### 2.2 pH homeostasis

Intracellular acidification due to accumulation of dissociated acetic acid in the cytosol can be counteracted by yeast cells through the activation of the plasma membrane H^+^‐ATPase (Pma1), which pumps protons out of the cell, and the vacuolar H^+^‐ATPase, which pumps protons into the lumen of the vacuole (Carmelo et al., 1997; Martínez‐Muñoz & Kane, 2008). Proton pumping is a high energy‐demanding process, consuming up to 20% of the cellular ATP produced in actively growing cells in the presence of glucose (Morsomme et al., 2000). Intracellular pH recovery due to Pma1p activity is a key determinant of acetic acid resistance, for which intracellular acidification is the major cause of growth inhibition (Ullah et al., 2012). This is also confirmed by the enhanced tolerance to acetic acid in *PMA1*‐overexpressing strains (Lee et al., 2017). Differently from other weak organic acids, acetate accumulation is not very toxic even at high concentrations and *PDR12*, responsible for acetate anions efflux, is not involved in acetic acid resistance (Ullah et al., 2012). This tolerance to anion accumulation can be explained by yeast capacity to produce and metabolise acetate at relatively high concentrations (Gancedo 1992). Also, *VMA3*, one of the major V‐ATPase assembly genes, contributes to acetic acid stress resistance by counteracting intracellular acidification and activating PKA signalling and glycolysis in the presence of glucose (Konarzewska et al., 2017).

### 2.3 Metabolism and stress‐signalling pathways

The threshold between tolerance and toxicity defines cell capacity to adapt or succumb to an environmental stress. At this regard, it is important to consider both mechanisms of tolerance (physiological state) and mechanisms of toxicity (pathological state) for a complete overview on acetic acid stress response. For acetic acid tolerance, a comprehensive picture focused on omics approaches is described in the work by Geng et al. (2017, and references therein). Omics studies revealed that acetic acid tolerance is controlled by multiple genes; the network of interaction among such genes is highly complex, and its elucidation is challenging even in a relatively simple model organism such as *S. cerevisiae*. Transcriptomic analysis identified Haa1p as the main player controlling yeast tolerance to acetic acid (Mira, Becker, & Sá‐Correia, 2010). On the other hand, the involvement in acetic acid tolerance of many genes related to carbohydrate metabolism, protein folding, lipid metabolism, cell wall function and transport has been elucidated by functional genomics screening and genome wide analysis (Mira, Palma, et al., 2010). Omics approaches could provide the theoretical basis and define genes for genetic modification in yeast, but the combination of methods able to capture complexity is necessary to obtain a clear picture of the cell‐wide acetic acid response in yeast. Therefore, high‐throughput techniques and systems metabolic analysis and advance genome scale modelling, also including regulation at certain levels, are needed for a deeper understanding of regulatory networks and, above all, their successful harnessing for cell factory development.

Most of the knowledge on the intracellular signalling of acetic acid toxicity comes from studies performed under extreme conditions affecting cell viability in different growth phases. Cell treatment of dividing cells with increasing concentration of acetic acid in the presence of glucose as sole carbon source at pH 3.0 is associated to forms of regulated cell death sharing apoptosis‐like (80–120 mM) or necrotic‐like (160–200 mM) features, respectively (Ludovico et al., 2001). Acetic acid concentrations higher than 120 mM are required to induce regulated cell death in stationary phase cells (Ludovico et al., 2002). Moreover, acetic acid deriving from ethanol under respiratory metabolism can mediate cell death through Sch9p and RAS/PKA pathway activation negatively affecting longevity (Burtner et al., 2009).

Overall, these evidences links acetic stress sensitivity to the environmental context, metabolic and energetic cellular profile. A strict relationship between carbon source and acetic acid tolerance is suggested by full resistance to acetic acid of dividing yeast cells grown on raffinose, but not on glucose, indicating that either intracellular metabolism or stress response pathways are activated under derepressing conditions (Guaragnella et al., 2013). In the detailed picture of cell components and mechanisms of yeast acetic acid‐induced regulated cell death (AA‐RCD), the pivotal role played by reactive oxygen species (ROS) and mitochondria emerge (Eisenberg et al., 2007; Guaragnella et al., 2012; Pereira et al., 2008). In particular, hydrogen peroxide appears to be a second messenger in the AA‐RCD cascade of events, as also shown by the inhibitory effect of the ROS scavenger N‐acetyl cysteine on AA‐RCD process (Guaragnella et al., 2010). Mitochondria play a dual role in AA‐RCD as for the release of apoptotic proteins (pro‐death), such as cytochrome *c*, as for the energy and metabolic supply (pro‐life). In a late phase of AA‐RCD, cytochrome *c* is degraded and mitochondrial dysfunction occurred with a decrease of the respiratory control index (RCI), a collapse of the mitochondrial membrane potential, a reduction in cytochrome *c* oxidase (COX) activity and in cytochromes a–a_3_ levels (Giannattasio et al., 2008; Ludovico et al., 2002). Thus, genetic or metabolic interventions to activate the antioxidant system or certain mitochondrial stress‐signalling pathways can counteract acetic acid toxicity. In this regard, low‐pH pre‐conditioning before acetic acid exposure or the overexpression of the cytosolic isoform of catalase, *CTT1*, also considered a general stress marker enzyme, has been shown to completely rescue AA‐RCD (Giannattasio et al., 2005; Guaragnella et al., 2008). The retrograde (RTG) response is a mitochondria‐to‐nucleus communication pathway for adaptation to mitochondrial dysfunction (Liu & Butow, 2006). Experimental evidences have shown that AA‐RCD resistance in raffinose grown exponential cells is partially due to the RTG pathway activation (Guaragnella et al., 2013; Ždralević et al., 2012). Interestingly, reduced oxidative stress and cyt *c* release together with simultaneous *SNF1*‐dependent relief of carbon catabolite repression have been observed in these cells, suggesting possible links between the central carbon metabolism reprogramming, mitochondria‐dependent AA‐RCD and the RTG pathways (Laera et al., 2016). This indicates that crosstalk between cell death and adaptation mechanisms are activated by acetic acid stress (Giannattasio et al., 2013; Sousa et al., 2013). The master regulator of transcription High‐osmolarity glycerol 1 (*HOG1*) is the main responsible of AA‐RCD resistance after low‐pH pre‐conditioning (Giannattasio et al., 2005; Guaragnella et al., 2019). Under these conditions, the RTG pathway is not activated but still involved with one of its positive regulator, Rtg2, acting as a general stress sensor and required for Hog1 activation (Guaragnella et al., 2019). Hog1 has also been reported to contribute to acetic acid resistance by inhibiting the acid entry through post‐translational regulation of the acetate channel Fps1 (Mollapour & Piper, 2007). Other pathways linked to RTG, TOR and Ras–cAMP–PKA, regulating nutrient sensing, metabolism, stress resistance and cell growth, are causally involved in the signalling of yeast AA‐RCD (Almeida et al., 2009; Giannattasio et al., 2013).

Several studies based on omics approaches, such as transcriptomics, proteomics and metabolomics, have been carried out to gain insight into the molecular determinants of acetic acid stress response. However, the different experimental settings, such as medium composition and pH, acetic acid concentration and duration of the treatment or cellular growth phase, do not always allow proper comparisons to obtain a homogenous picture. In addition, a strain‐dependent tolerance to acetic acid has to be also considered (Table 1).

**TABLE 1:** Acetic acid concentrations, pH and the resulting undissociated acetic acid concentrations in the cited studies

Reference	Strain background	Acetic acid (mM)	Acetic acid (g/l)	pH
Almeida et al. (2009)	BY4742	140–200	8.4–12.0^a^	3.0
Ask et al. (2013)	CEN.PK 113‐7D	55^a^	3.3	5.0
Burtner et al. (2009)	BY4742	10	0.6^a^	2.5
Casal et al. (1996)	IGC4072	87^b^	5.2^b^	5.0
Y. Chen et al. (2016)	D452‐e2	182^a^	10.9	4.0
C. Chen et al. (2016)	BY4741	92^a^	5.5	4.0, 4.8
Cunha et al. (2018)	PE‐2	73^a^	4.4	4.0
Ding et al. (2015)	S288C	140	8.4^a^	4.8
Dong et al. (2017)	W303	30–150	1.8–9.0^a^	3.0
Giannattasio et al. (2008)	W303	20–80	1.2–4.8^a^	3.0
Giannattasio et al. (2005)	W303	20–200	1.2–12.0^a^	3.0
Godinho et al. (2018)	BY4741	50–100	3.0–6.0^a^	4.0
Guaragnella et al. (2008)	W303	80	4.8^a^	3.0
Guaragnella et al. (2010)	W303	20–200	1.2–12^a^	3.0
Guaragnella et al. (2006)	W303	80	4.8^a^	3.0
Guaragnella et al. (2019)	W303	80	4.8^a^	3.0
Guaragnella et al. (2013)	W303	80	4.8^a^	3.0
Gurdo et al. (2018)	BAFC 3084	83^a^	5.0	6.0
Hu et al. (2019)	BY4742	150	9.0^a^	3.0
Kim et al. (2019)	BY4741	30	1.8^a^	7.4
Kitanovic et al. (2012)	FF 18984	30–120	1.8–7.2^a^	3.0–7.0
Konarzewska et al. (2017)	BY4741	25, 50	1.5, 3^a^	N/A
Laera et al. (2016)	W303	80	4.8^a^	3.0
Lee et al. (2017)	BY4741	105^b^	6.3^b^	4.5
Lindberg et al. (2013)	CEN.PK 113‐7D	30–200^a^	1.8–12.0	5.0
Longo et al. (2015)	W303	80	4.8^a^	3.0
Ludovico et al. (2001)	W303	20–200	1.2–12.0^a^	3.0
Ludovico et al. (2002)	W303	120–240	7.2–14.4^a^	3.0
Meijnen et al. (2016)	Ethanol Red, JT22689	110–167^b^	6.6–10.0^b^	4.0
Mira, Palma, et al. (2010)	BY4741	60	3.6^a^	4.0
Mira, Becker, and Sá‐Correia (2010)	BY4741	70–110	4.2–6.6^a^	4.5
Mollapour and Piper (2007)	BY4741	100	6.0^a^	4.5
Oh et al. (2019)	CEN.PK2‐1D	83^a^	5.0	4.0
Pampulha and Loureiro‐Dias (1989)	IGC 3507 III	40–200	2.4–12^a^	3.5–5.5
Raghavendran et al. (2020)	CEN.PK 113‐7D	55^a^	3.3	5.0
Sousa et al. (2013)	BY4741	400	24^a^	3.0
Swinnen et al. (2017)	BY4741	200	12^a^	4.5
Tanaka et al. (2012)	S288C	167^a^	10.0	4.2
Ullah et al. (2012)	BY4741	42	2.5^a^	5.0
Wu et al. (2016)	BY4741	75^a^	4.5	4.0
Zhang et al. (2017)	BY4741	60–167^a^	3.6–10	4.5
Zhang et al. (2015)	BY4741	7^a^	4.3	4.8

*Note*: Values separated by ‘–’designate a range, and values separated by commas designate the specific values used.

Abbreviation: N/A, not available.

^a^ Recalculated from grams per litre or millimolar. When values in millimolar were given, the corresponding concentration in grams per litre has been recalculated and vice versa.

^b^ Recalculated from % v/v.

In AA‐RCD regulation, the yeast metacaspase Yca1 has been shown to play an extensive role causing significant alterations in carbohydrate catabolism, lipid metabolism, proteolysis and stress‐response as judged by proteome and metabolome profiling of *YCA1*‐knock out cells during AA‐RCD (Guaragnella et al., 2006; Longo et al., 2015). Particularly, a shift from the main glycolytic pathway to the pentose phosphate pathway and a proteolytic mechanism to cope with oxidative stress characterise AA‐RCD in the presence of *YCA1*, while AA‐RCD occurs through the activation of ceramide metabolism in its absence (Longo et al., 2015). It is of note that metacaspases are ancestors of mammalian caspases and their extensive biological functions reflects the involvement in a regulated death process occurring in a context of a failing response to internal or external mild stress. In this regard, obvious differences exist between mammalian apoptosis and yeast regulated cell death in terms of specific morphologic and biochemical features, effectors and key molecular players (Carmona‐Gutierrez et al., 2018; Kulkarni et al., 2019).

For the modulation of AA‐RCD in yeast, the relevance of certain genes, especially involved in mitochondrial function, glucose repression and oxidative stress response, has been confirmed at genome‐wide scale (Sousa et al., 2013). The work by Almeida et al. (2009) identifies TOR pathway as an important regulatory node during a late phase of AA‐RCD and indicates that the proteomic alterations are directly or indirectly linked to a TOR‐dependent regulation and include amino acid uptake, transportation and synthesis (Almeida et al., 2009). That acetic acid affects amino acids transport, particularly between cytosol and vacuole, has been recently confirmed by a study on knock out yeast cells lacking the vacuolar autophagy‐related protein ATG22 (Hu et al., 2019).

A transcriptomic and metabolomic analysis show temporal‐ and spatial‐specific expression in acetic acid treated cells, implying the upregulation of genes involved in transcription and protein synthesis in an early‐phase; protein fate, cell cycle and DNA processing in a middle stage and regulation of metabolism and protein function in a late phase. Meanwhile, genes involved in cellular transport, transport facilities and transport routes were reduced in both early and middle stage (Dong et al., 2017).

## 3 STRATEGIES TO IMPROVE ACETIC ACID TOLERANCE

Acetic acid is an important source of stress and a potent inhibitor during yeast‐based industrial fermentation processes, such as wine making and production of fuels and chemicals from renewable carbohydrate feedstocks, including lignocellulosic biomass. This latter procedure involves preliminary pretreatment and hydrolysis steps to convert polysaccharides into sugar monomers, which are then converted to produce desired products. Acetic acid is the main by‐product of both lignocellulose pretreatment and fermentation process, inhibition affects productivity and growth of *S. cerevisiae* and the capacity of fermenting alternative yet abundant carbon sources, such as xylose in recombinant strains. For this reason, implementing efficient and economical strategies to obtain robust cell factories and increase acetic acid resistance represent a critical challenge for commercial production of cellulosic fuels and chemicals. Numerous studies and different approaches, mostly developed in laboratory *S. cerevisiae* strains, allowed to identify important factors, components and regulatory networks for constructing more robust industrial yeast strains to be used in the field of ethanologenic fermentation. A list of genes involved in acetic acid tolerance and identified through different methodological approaches has been reported in Table 2.

**TABLE 2:** Genes involved in acetic acid tolerance

Gene	Function	Acetic acid tolerance	Methodology	Strain background	Reference
FPS1	Aquaglyceroporin	**+**	Deletion	BY4741	Mollapour and Piper (2007)
ADY2	Acetate transporter	**+**	Deletion	BY4741	Zhang et al. (2017)
PDR18	Transporter of ABC family	**−**	Deletion	BY4741	Godinho et al. (2018)
PMA1	Plasma Membrane H+‐ATPase	**+**	Overexpression	BY4741	Lee et al. (2017)
VMA3	Vacuolar H+‐ATPase	**−**	Deletion	BY4741	Konarzewska et al. (2017)
HAA1	Transcriptional activator for weak acid stress	**+**	Deletion/Transcriptomics Overexpression	BY4741 S288C	Mira, Becker, and Sá‐Correia (2010) and Tanaka et al. (2012)
RTG2	Sensor of mitochondrial dysfunction	**−**	Deletion	W303	Guaragnella et al. (2013)
HOG1	Mitogen‐activated protein kinase	**−**	Deletion	W303	Guaragnella et al. (2019)
RCK1	Protein kinase	**+**	Overexpression	D452‐2	Oh et al. (2019)
WHI2	Protein phosphatase activator	**+/−**	Overexpression Deletion	D452‐2	Chen et al. (2016)
SET5	Methyltransferase	**+**	Overexpression	BY4741	Zhang et al. (2015)
PPR1	Zinc finger transcription factor	**+**	Overexpression	BY4741	Zhang et al. (2015)
CTT1	Cytosolic catalase	**+**	Overexpression	W303	Guaragnella et al. (2008)
JJJ1	ATPase activator	**+**	Deletion	BY4741	Wu et al. (2016)
RTT109	Histone acetyltransferase	**+**	Deletion	BY4741	Cheng et al. (2016)
ACS1	AcetylCoA‐synthetase 1	**+**	Overexpression	XM19/XM20	Ding et al. (2015)
ACS2	AcetylCoA‐synthetase 2	**+**	Overexpression	S288C	Qin et al. (2020)

To enhance the competitiveness of industrial lignocellulosic fuels and chemicals, robust enzymes and cell factories are vital. Lignocellulose‐derived streams contain a cocktail of inhibitors, including acetic acid, that drain the cell of ATP and redox potential, causing a multi‐level cell response. Also in this case, results from applied research for cell factory development point to the fact that there is no ‘magic bullet’ and targeting a single gene, without taking into consideration a cell‐wide response, often results in unforeseen cell response, counteracting or neutralising the expected potential beneficial effects.

### 3.1 Overexpression or deletion of individual genes

Although studies on the effects of overexpression or deletion of single genes on acetic tolerance differ for strains and experimental settings, they mostly converge on common defence mechanisms, involving general stress response, particularly oxidative stress, metabolic and energetic aspects. Overexpression of *RCK1*, coding for a protein kinase, has been recently reported to confer higher resistance to acetic acid by reducing oxidative stress (Oh et al., 2019). This is in accordance with the proposed mechanism for Whi2, whose overexpression could activate the transcription factors Msn2/Msn4 and consequently the expression of stress response genes related to acetic acid tolerance (Chen et al., 2016). The strict link between oxidative stress and acetic acid resistance is also confirmed by the overexpression of *SET5* and *PPR1* exerting their functions in the presence of acetic acid through both global gene transcription and metabolic regulation via ROS detoxification upon a pH of 3.5 (Zhang et al., 2015). At this regard, a specific role for the antioxidant enzyme catalase has been related to increased acetic acid tolerance either in *CTT1* overexpressing cells or in *JJJ1* and *RTT109* knockout cells (Cheng et al., 2016; Guaragnella et al., 2008; Wu et al., 2016). These evidences reinforce the concept that the maintenance of intracellular redox balance and particularly detoxification of H_2_O_2_ and not O_2_˙ is a key condition for multiple stress tolerance, including acetic acid, in *S. cerevisiae* (Gurdo et al., 2018). Also, the deletion of the plasma membrane acetate transporter *ADY2* improves growth and fermentation under acetic acid stress, by reducing ROS accumulation and increasing cell membrane integrity (Zhang et al., 2017). Increasing cell capacity to consume acetic acid through the overexpression of acetyl‐coenzyme A synthetases 1 or 2, *ACS1* or *ACS2*, is another possible strategy for improving acetic acid tolerance by accelerating carbon and energy metabolism (Ding et al., 2015; Qin et al., 2020).

Interestingly, a beneficial effects of flocculin genes and zinc supplementation on growth and fermentation capacity has been revealed in the development of acetic acid tolerant *S. cerevisiae* strains by metabolic engineering (Cheng et al., 2017, and references therein). Upon zinc supplementation and acetic acid stress, a protective effect has been observed after overexpression of *ADE* genes enhancing cell growth, improving ethanol productivity, controlling purine and amino acid biosynthesis (Zhang et al., 2017).

### 3.2 Manipulation of Haa1‐Regulon

Another possibility to improve strain robustness against acetic acid is acting on the Haa1‐regulon. The major role played by this transcription factor in acetic acid stress has been largely demonstrated at genomic, genetic and molecular level (Kim et al., 2019; Meijnen et al., 2016; Mira, Becker, & Sá‐Correia, 2010). Haa1 overexpression itself or in combination with the overexpression of the phosphoribosyl pyrophosphate synthetase encoded by PRS3 in a recombinant industrial *S. cerevisiae* strain boosts yeast tolerance towards acetic acid (Cunha et al., 2018; Tanaka et al., 2012). The known mechanisms of Haa1‐mediated acetic acid stress response involve the cell wall integrity pathway and the activation of plasma membrane multidrug transporters to reduce intracellular acetate accumulation (Cunha et al., 2018; Swinnen et al., 2017). Also, the phosphorylation state of Haa1 can affect its transcriptional activity and consequently stress response to acetic acid (Collins et al., 2017).

### 3.3 Synthetic biology and metabolic engineering approaches

The ‘omics’ era has offered a new opportunity and perspective in the comprehension of biological processes and allowed the development of synthetic biology and metabolic engineering approaches. Also, the knowledge on acetic acid tolerance has been extended to a genomic point of view and important achievements based on the manipulation of genomes or metabolic fluxes have been reached to counteract acetic acid toxicity (Palma et al., 2018). An industrial *S. cerevisiae* strain tolerant to high concentrations of acetic acid and with improved performance during 2G ethanol production was obtained through the evolutionary adaptation strategy of metabolic engineering conferring physiological or genotype variations, specifically higher oxidative stress resistance (Gurdo et al., 2018). Evolutionary engineering combined with genome shuffling via mating allowed to generate recombinants *S. cerevisiae* strains with improved tolerance to key inhibitory by‐products present in hydrolysates of lignocellulosic biomass, including acetic acid and to identify the specific mutations responsible for the improved fitness by whole‐genome resequencing (Cheng et al., 2015). Transcriptome profile of cells exposed to acetic acid under various conditions and different times confirmed that enhanced acetic acid tolerance is attributed to multiple factors. At this regard, an engineered *S. cerevisiae* strain with improved xylose utilisation and ethanol production has been obtained by creating a feedback regulation system through the simultaneous overexpression of selected genes regulated by stress‐driven promoters and strengthening the GSH (glutathione) biosynthesis and acetate degradation pathways (Qin et al., 2020). This is consistent with other observations, demonstrating the higher overall tolerance to lignocellulosic hydrolysates of GSH‐accumulating strains (Ask et al., 2013).

Notwithstanding the positive effect on enhanced GSH accumulation, such multiple overexpression system may in themselves become the cause of cell stress, causing metabolic burden and unforeseen cells responses, as shown by recent results obtained with recombinant strains accumulating high levels of GSH (Raghavendran et al., 2020).

## 4 CONCLUSIONS

**FIGURE 1:**
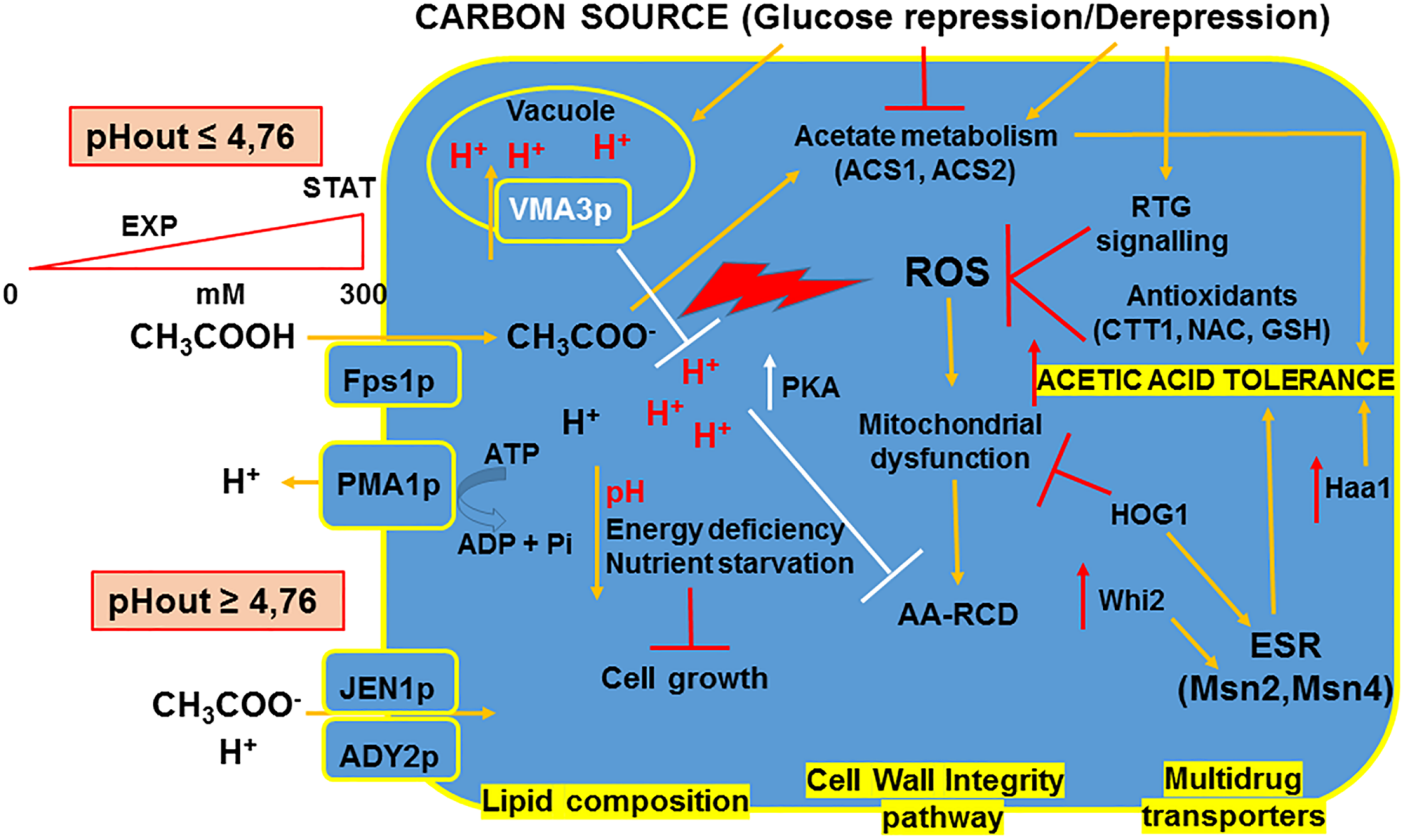
Major factors and cell components involved in acetic acid stress and its response in budding yeast. Extracellular and intracellular pH, cellular growth phase and environmental growth conditions, plasma membrane composition and cell wall assembly, activity of plasma membrane/vacuolar proton pumps and monocarboxylate transporters, metabolic and antioxidant genes, antioxidant molecules, transcription factors and stress‐signalling pathways [Colour figure can be viewed at wileyonlinelibrary.com]

Reflecting on the molecular mechanisms of acetic acid stress response in budding yeast gives the opportunity to get an integrated view connecting aspects related to cellular transport, pH homeostasis, metabolism and stress‐signalling pathways (Figure 1). All this knowledge supports the relevance of an environment‐dependent cell fitness and adaptation under acid stress conditions. Integrating this information with the results obtained by synthetic biology and metabolic engineering approaches in the ‘omics’ era is a valuable resource in industrial biotechnology, where the improvement of acetic acid tolerance is a key factor during yeast‐based fermentation processes. In this view, the production of biofuels and renewable chemicals from lignocellulosic biomass represents one of the most promising goals to promote a sustainable circular and biobased economy. In order to develop the performing cell factories required for the competitiveness of this sector, the knowledge on the single molecular signalling pathways that has accumulated over the years, as well as the current capacity of developing system‐wide approaches for modelling and engineering cell pathways have to come into play.
